# Topical Povidone Iodine 2.5% Versus 5% for Endophthalmitis Prophylaxis During Intravitreal Injections

**DOI:** 10.1155/joph/6629976

**Published:** 2025-10-30

**Authors:** Radwan S. Ajlan, Shreeya Dalla, Riya J. Parikh, Matthew M. Pfannenstiel, Mary T. Champion, Md Atikur Rahman, Francisco J. Diaz

**Affiliations:** ^1^Department of Ophthalmology, University of Kansas School of Medicine, Kansas City, Kansas, USA; ^2^Department of Biostatistics and Data Science, University of Kansas Medical Center, Kansas City, Kansas, USA

## Abstract

**Purpose:**

Topical povidone iodine (PI) has been shown to reduce the risk of endophthalmitis following intravitreal injections (IVIs). However, PI is a known ocular irritant and can result in significant eye discomfort. Currently, 5% PI is the most used concentration for ocular surface disinfection prior to IVI. 2.5% PI has been proposed as an alternative IVI preparation to lessen PI-associated ocular irritation. The purpose of this study is to compare the efficacy of topical PI 2.5% to PI 5% in preventing post-IVI endophthalmitis at a single academic institution location.

**Methods:**

A retrospective chart review was conducted at a single academic institution location of patients receiving IVI from August 1st, 2017, to June 15th, 2022. IVIs were performed using PI 2.5% or PI 5%. PI 5% was applied to the ocular surface 60 s before IVI. PI 2.5% was applied over the ocular surface and then reapplied 4-5 min later over the injection site and fornices before IVI.

**Results:**

A total of 7360 IVIs were performed in 773 patients (473 patients on 2.5% and 300 on 5% PI). 52.0% of IVIs were performed with 2.5% PI (*n* = 3826) and 48.0% were performed with 5% PI (*n* = 3534). The mean age of patients was 66.8 years for the 2.5% PI group and 69.1 years for the 5% PI subgroup (*p*=0.044). Three cases of endophthalmitis were identified in the 5% PI subgroup (0.08%), and no cases of endophthalmitis were identified in the 2.5% PI subgroup. All cases of endophthalmitis were treated with prompt intravitreal antimicrobial injection.

**Conclusion:**

In this retrospective study, we found that 2.5% and 5% PI had similar results in preventing post-IVI endophthalmitis. This study adds to the literature by further supporting the antiseptic effect of 2.5% PI for IVI. Larger prospective studies are needed to further clarify the antiseptic effect and ocular surface irritation associated with 2.5% PI use.

## 1. Introduction

Since its introduction in the 1950s [1], the water-soluble disinfectant povidone iodine (PI) has been commonly used to prevent wound infections, with no reported cases of resistance [2]. PI has been used since the 1980s to prevent infection in ophthalmic surgeries [3]. Its preoperative topical application has become the standard of care for intraocular surgery, including cataract extraction and pars plana vitrectomy [4]. For intravitreal injections (IVIs) in particular, topical application of PI has become an essential step during IVI antisepsis [4–6].

IVI is used to administer medications, such as antivascular endothelial growth factors (antiVEGFs) therapy and steroids, to the posterior segment of the eye. IVI has become one of the most common intraocular procedures performed by ophthalmologists and has been very successful at treating a variety of retinal pathologies, such as age-related macular degeneration, diabetic macular edema, and retinal vascular occlusions [7–9]. During the IVI injection procedure, PI is applied topically to the injection site.

PI is composed of povidone (polyvinylpyrrolidone) and iodine. Povidone acts as a water-soluble polymer to carry iodine, which has long been used as a disinfectant. PI is an intermediate-level disinfectant that can be used on skin and ocular tissue when diluted to an appropriate concentration. It is effective against multidrug-resistant bacteria, fungi, and hepatitis virus, but not spores [1]. Although the mechanism of PI sterilization is not well understood, studies have shown that it decreases the risk of bacterial, fungal, and viral infections without conferring resistance.

One drawback of PI is that it stimulates the ocular surface, which can lead to eye irritation following its application. This discomfort may be reduced by lowering the concentration of PI. Lower concentrations of PI (0.1%–1.0%) have higher bactericidal activity but a shorter duration of action because of lower iodine reserves. Thus, multiple PI applications are necessary with lower concentrations. In contrast, higher concentrations of PI (2.5%–10%) require longer contact duration with the target tissue and have a longer residual effect, due to higher iodine reserves [1–3]. Topical antibiotics and chlorhexidine are commonly used instead of PI, but both have disadvantages. Topical antibiotics increase the risk of antibiotic-resistant organisms and have not been shown to decrease the risk of post-IVI endophthalmitis [1]. Topical chlorhexidine gluconate 0.1% has better tolerability than PI but a narrower antimicrobial spectrum [10].

Conjunctival lavage in cornea donors found no difference in colony forming units after 2 minutes of exposure of PI 1% compared to 2 minutes of exposure of PI 5%; however, PI 5% caused higher corneal toxicity [11]. Based on the available literature, we hypothesized that PI 2.5% may effectively prevent post-IVI endophthalmitis, provide abundant iodine for residual effect after application, and result in less cornea surface toxicity than higher doses. Currently, the effect of PI 2.5% in post-IVI endophthalmitis prophylaxis is not well established. To address this gap in knowledge, we performed a retrospective study to compare the efficacy of topical PI 2.5% to PI 5% in preventing post-IVI endophthalmitis at a single academic institution location.

## 2. Methods

A retrospective chart review was conducted at a single academic institution location. Inclusion criteria comprised all adult patients who had IVI from August 1st, 2017, to June 15th, 2022. The study excluded pediatric population and patient who had IVI outside the study period. During the study period, IVIs were conducted by two vitreoretinal specialists at the institution. Collected data included patients' demographics, IVI medication, underlying condition requiring IVI, and cases of endophthalmitis. Institutional Review Board approval was obtained.

### 2.1. IVI Procedure

The same IVI procedure protocol and “no talk policy” was injection were used during injection for both study groups, with minor differences. In the PI 5% group, PI drops were applied topically over the injection quadrant and IVI was performed 60 s afterward. In the PI 2.5% group, PI drops were applied over the ocular surface and reapplied four to 5 minutes later over the injection site and fornices before IVI was performed. For the PI 5% group, nonsterile medical gloves were used, and for the PI 2.5%, sterile medical gloves were used. As a result of the COVID-19 pandemic, a face mask mandate was established during the study period, from April 4^th^, 2020, to June 15^th^, 2022. During this period, all staff and patients were required to wear masks. Charts were reviewed for the number of cases of endophthalmitis before and after the mask mandate was established.

### 2.2. Biostatistics

Mean total number of IVIs was calculated for the 2.5% and 5% PI groups stratifying by product. This study included patients who had only one of these two doses in all visits. A negative binomial regression model of the total number of Aflibercept IVIs was fitted. The dependent variable of this model was essentially the logarithm of the number of injections of a patient, after dividing it by the number of days during which the recorded injections were administered to the patient (the patient's observation time). As model covariates, the model included age, gender, and PI dose, having a diagnosis of diabetic retinopathy and having a diagnosis of macular degeneration. The observation time was entered as a logged-offset variable. The mean of the ratio of the number of injections to the observation time is the injection rate. The model provides injection rate ratios. An injection rate ratio corresponding to a binary variable compares the injection rates of the two categories represented by the variable. Similar models were fitted for Bevacizumab and Ozurdex. No model was fitted for Ranibizumab because of the relatively small number of IVIs (146). An additional model of the total number of IVIs regardless of product was fitted. We also tested interactions between covariates in this model.

Of the 773 patients investigated, 340 were treated in their two eyes (44%). These subjects provided data from the two eyes, which produced correlated data. The negative binomial regression models included a random intercept to account for the fact that each patient had their own intercept in the linear relationship between the log of the injection rate and the covariates and the fact that each patient provided more than one observation. A *p* value of 0.05 was considered statistically significant. The statistical software Stata was used for analyses, implementing the meglm command (StataCorp LLC, College Station, TX).

## 3. Results

Over the 5 year study period, a total of 7360 IVIs were performed in 773 patients (473 patients on 2.5% and 300 on 5% PI). 52.0% of IVIs were performed with 2.5% PI (*n* = 3826) and 48.0% were performed with 5% PI (*n* = 3534). The mean age of patients was 66.8 years in the 2.5% PI group and 69.1 years in the 5% PI group (*p*=0.044). The 2.5% PI group was 49.3% male, and the 5% PI group was 46.0% male (*p*=0.38).  Table 1 describes the patients' demographics and most comment underlying ocular pathology. Endophthalmitis occurred in three patients following IVI, an overall incidence of 0.04%. All three cases of endophthalmitis occurred in the 5% PI group (0.08%), and zero cases occurred in the 2.5% PI group (0%). The difference was not significant (Fisher exact *p*=0.10). All three cases of endophthalmitis occurred prior to the onset of the masking protocol (*p* > 0.05) and were treated with injection of vancomycin (1 mg/0.1 mL), ceftazidime (2.25 mg/0.1 mL), and voriconazole (400 μg/0.05 mL) through the pars plana. 5% PI and speculum insertion were utilized prior to treatment injections, and vitreous tap was performed for culture. Vitreous cultures grew *Staphylococcus epidermidis* in one patient, and the remaining two patients had no bacterial or fungal culture growth. Mean time between IVI and presentation in clinic for endophthalmitis was 1.3 days.

**Figure 1:**
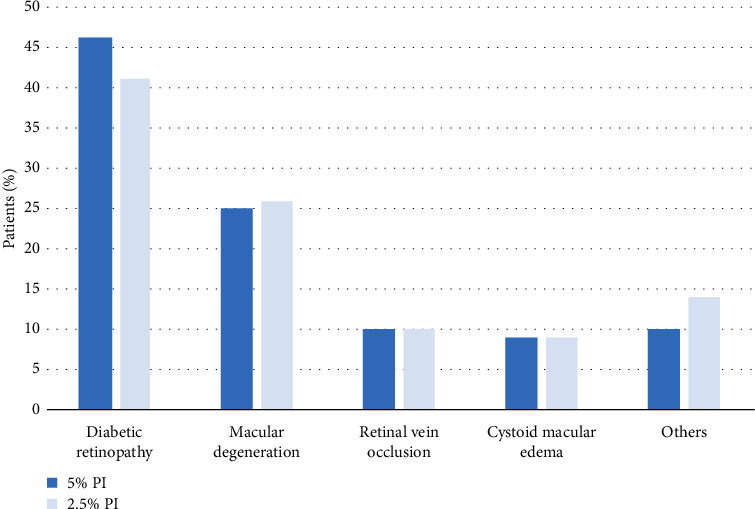
Indications for intravitreal injection in the study cohort. Majority of the patients needed intravitreal injections for diabetic retinopathy, followed by wet macular degeneration.

The most common indication for IVI in both the 2.5% and 5% PI groups was diabetic retinopathy (41% and 46%, respectively), followed by macular degeneration (26% and 25%, respectively) (Figure 1). The most common medication to be injected in both the 2.5% and 5% PI groups was aflibercept (69% of IVIs [5099/7360]).

Before adjusting for patient's observation time, the 5% PI group had a significantly higher mean number of aflibercept, bevacizumab, and Ozurdex injections compared to the 2.5% PI group (*p* < 0.05, Table 2). However, the difference between PI doses was no longer significant after adjusting for observation time (Table 2). For ranibizumab, no significant differences in the mean number of IVIs were observed between PI groups, both before and after adjusting of observation time.

Tables 3, 4, and 5 show unadjusted and adjusted effects of potential confounders on the number of aflibercept, bevacizumab, and ozurdex injections, respectively. The negative binomial regression model showed the aflibercept injection rate significantly increased with age after adjusting for patient's observation time, gender, PI dose, diabetic retinopathy, and macular degeneration diagnoses. The rate increased 2.2% for each year of age [adjusted rate ratio, 1.022; 95% CI (1.013, 1.030); *p* < 0.001, Table 3]. In contrast, the bevacizumab injection rate significantly decreased with age [adjusted rate ratio, 0.974; 95% CI (0.963, 0.984); *p* < 0.001; Table 4].

After adjusting for potential confounders, the rate of aflibercept injections under the 2.5% dose was 19.9% significantly lower than that under the 5% dose (adjusted rate ratio of 0.801; 95% CI (0.660, 0.972); *p*=0.025; Table 3). In contrast, we did not observe significant differences between PI doses of bevacizumab and Ozurdex (Tables 4 and 5).

After adjusting for potential confounders, patients with diabetic retinopathy received aflibercept injections at a significantly higher rate than patients without diabetic retinopathy (adjusted rate ratio of 2.081; *p* < 0.001, Table 3). A similar result was observed for Bevacizumab (Table 4). But the opposite result was observed for Ozurdex: patients with diabetic retinopathy had Ozurdex injections at a significantly lower rate than patients without diabetic retinopathy (adjusted rate ratio of 0.264; *p* < 0.001, Table 5). Interestingly, the effects of macular degeneration were analogous. Patients with macular degeneration had significantly higher rates of aflibercept injections (*p* < 0.001, Table 3) and higher rates of bevacizumab injections (*p*=0.001, Table 4) than patients without macular degeneration, but they had lower rates of Ozurdex injections (*p* < 0.001, Table 5). After adjusting for potential confounders, gender did not have a significant effect on the number of aflibercept (Table 3), bevacizumab (Table 4), or Ozurdex (Table 5) injections.

We did not have information on whether aflibercept, bevacizumab, Ozurdex, or ranibizumab injections occurred before or after the COVID-19 pandemic. We operationally defined the initiation of the pandemic at April 9, 2020. However, we were able to assess when the overall treatment started for the patient. We fitted a negative binomial regression of number of injections regardless of product, adjusting for potential confounders including an indicator of whether the COVID-19 pandemic started before or after the initiation of the pandemic (Table 6). Before adjusting for potential confounders, the COVID-19 pandemic significantly reduced the rate of injection applications (unadjusted injection rate ratio of 0.853, *p*=0.006). However, after adjusting for confounders, we observed that there was a significant interaction between gender and the occurrence of the COVID-19 pandemic (*p*=0.003, Table 6). Specifically, we observed that, in males, the IVI rate was 26.7% lower during the COVID-19 pandemic compared to before the COVID-19 pandemic [adjusted rate ratio of 0.732; 95% CI (0.627, 0.855)], but the COVID-19 pandemic did not significantly change the IVI rate in females [adjusted rate ratio of 1.009; 95% CI (0.867, 1.174), Table 6]. Finally, there was a borderline significant interaction between PI dose and age, suggesting that for each year of age, the injection rate ratio for the 2.5% PI dose compared to the 5% dose increased by 0.8%, implying that the lower dose tended to be more frequent in older people (Table 6). After controlling for potential confounders, the side of the treated eye (left or right) did not have any significant effect on the number of injections administered (data not shown).

## 4. Discussion

In this retrospective review, we observed no cases of IVI-related endophthalmitis in patients treated with 2.5% PI and three cases in the 5% PI group. This difference was nonsignificant, indicating that 2.5% PI was not inferior to 5% PI as an IVI-related endophthalmitis prophylaxis. For patients who did have endophthalmitis, vitreous cultures were positive for *Staphylococcus endophthalmitis* in one patient and showed no growth in the other two patients.

IVI is the most commonly performed eye procedure worldwide and has changed retinal disease management and restored vision to many patients [12]. IVI-related endophthalmitis is a sight threatening complication with a prevalence of 0.015%–0.083% with anti-VEGF therapy [7–9, 12]. During procedures which penetrate the eye, endophthalmitis commonly occurs due to the introduction of ocular surface pathogens inside the eye [13]. Topical PI application is a key factor in decreasing the risk of IVI-related endophthalmitis. The American Academy of Ophthalmology (AAO) recommended applying PI over the injection site immediately prior to injection, referencing studies finding similar efficacy of 5% PI compared to 10% PI, with less ocular irritation [6]. A Euroretina consensus, published in 2018, recommended applying 5% PI topically for at least 30 s into the conjunctival sac.

The microbicidal effect of PI comes from iodine, which oxidizes water to release ions that act on bacterial or viral membrane proteins, as well as on human cell membrane proteins. PI concentration and time of exposure have a major role in the initial and residual effect of iodine. Different PI concentrations differ in their concentrations of free iodine. Free iodine concentration is 13 ppm in 0.01% PI, 24 ppm in 0.1% PI, 13 ppm in 1% PI, and 5 ppm in 10% PI. Thus, lower concentrations of PI have higher concentrations of free iodine and therefore require a shorter time to effect sterilization: 15 s for 0.1%–1% PI and 30–120 s for 2.5%–10% PI. When PI is applied, the available free iodine is consumed and rendered inactive, necessitating replenishment from iodine reserves. The concentration of iodine reserves differs between PI concentrations. 0.1%–1.0% PI have lower iodine reserves and therefore require multiple applications, compared to 2.5%–10% PI, which have abundant iodine reserves and only require a single application [5].

Clinically, a 15 s exposure to 5% PI had limited effect on bacterial CFU, and thus a minimum exposure time of 30 s is advised prior to IVI [4, 14]. A study of bacterial endophthalmitis isolates on donor corneoscleral tissue found that 5% PI was comparable to 2.5% PI in preventing bacterial growth after 3 min of exposure; however, this was not true in the case of *Streptococcus viridines*, where 5% PI was more effective [15].

Worldwide, the recommended PI exposure time varies. In the US, a minimum of 30 s exposure time is recommended prior to IVI. In France, a two-minute exposure is recommended, and in the UK, 3 min is recommended [16]. In our study, topical 5% PI was applied over the injection site a minimum of 30 s before IVI, while 2.5% PI was applied before subconjunctival anesthesia, allowing a four to 5 min exposure time, and PI was applied again to the injection site and fornices immediately before IVI.

Ocular surface toxicity increases as the duration of PI contact increases and as the concentration of PI increases. A study in rabbit eyes found higher epithelium toxicity with 5% PI compared to 2.5% PI or lower. Pain after topical PI application is frequently caused by ocular surfaces exposure to PI, especially with 5%–10% PI [4]. Ikuno et al. reported ocular irritation in patients following 5% PI application but no ocular irritation in patients following 1.25% PI and 0.5% topical levofloxacin application [17]. Similarly, Peden et al. found that patient discomfort was significantly higher with 5% PI than with diluted PI concentration [18]. In this study, to minimize post-procedure discomfort, 5% PI was applied to the inferior-temporal conjunctival quadrant immediately prior to IVI, while 2.5% PI was applied to the injection site and conjunctival fornices. Our study did not evaluate differences in ocular surface irritation between 2.5% PI and 5% PI. However, when 2.5% PI became unavailable at our institute, many patients in the 2.5% PI group reported increased ocular surface irritation when they were switched to 5% PI.

While the current standard of care for IVI is 5% PI, many ophthalmologists have taken a significant interest in lower-concentration PI, as it has the potential to improve patients' experience and increase adherence to follow-up by reducing discomfort. Kaymaz et al. reported nine cases of endophthalmitis following a total of 15,345 IVI injections with the application of 0.25% PI. However, the study used sterile draping, and the physician, nurse, and patient wore bonnets, masks, and shoe-covers before entering the injection room [19]. Similarly, an earlier study from Japan found significantly low rates of endophthalmitis after performing 15,144 injections when applying 0.25% PI and using sterile draping, masks, and caps [20]. While these studies provide significant data on reduced risk of endophthalmitis with lower PI concentration, this is not necessarily replicable at all institutions. Sterile draping and sterile gloves are not the standard practice for IVI, and not all institutions have the resources to perform such practices. Sterile gloves use in the PI 2.5% group was the injecting physician's preference, and we reported it to avoid any bias. In our study, the two main procedure differences between groups were the PI application duration and location to the conjunctival fornices in the PI 2.5% group.

The most common pathogen associated with IVI-related endophthalmitis is coagulase-negative *Staphylococci*, specifically *Staphylococcus epidermidis* [21], with the second most common being *Streptococcus viridians* [22]. Microbial culture yield in the literature varies from 18% to 64% [23]. In our study, cultures were taken from patients with suspected endophthalmitis; one culture was positive, with a microbial yield of 33%, and two cultures were negative. The culture-positive sample grew *Staphylococcus epidermidis* and was susceptible to vancomycin.

During the COVID-19 pandemic, we found a significant decrease in the rate of injections per day, likely reflecting the pandemic impact on sight-saving IVI retina clinic appointments. Universal face masking was mandated for health care teams and patients during the pandemic. All three endophthalmitis cases occurred before the pandemic. The rate of endophthalmitis was very low before the pandemic and was statistically insignificant when compared to the endophthalmitis rate during the pandemic, which correlates with the current literature. Both cohorts used a no talking approach when performing IVI before the pandemic.

We recognize several limitations to this study. Retrospective chart reviews are by their nature inferior to randomized controlled studies. Additionally, this study did not evaluate the patients' reports of eye irritation postinjection, and future studies are needed to determine if 2.5% PI significantly reduces ocular discomfort in patients who are sensitive to 5% PI. Another limitation in this study is the different duration and location of PI application between groups.

## 5. Conclusion

In this retrospective study, we found that topical PI 2.5% use before IVIs had similar results in preventing post-IVI endophthalmitis as topical 5% PI. This study adds to the literature by further supporting the antiseptic effect of 2.5% PI for IVIs.

## Figures and Tables

**Table 1:** Description of investigated patients.

Variables	PI dose			*p* value
	All (*N* = 773)	2.5% (*N* = 473)	5% (*N* = 300)	
Age (mean ± SD)	67.7 ± 15.7	66.8 ± 15.2	69.1 ± 15.9	0.044^a^
Gender				
Male (%)	371 (48.0%)	233 (49.3%)	138 (46.0%)	0.38^b^
Female (%)	402 (52.0%)	240 (50.7%)	162 (54.0%)	
Diabetic retinopathy				
Yes	362 (46.8%)	253 (53.5%)	109 (36.3%)	< 0.001^b^
No	411 (53.2%)	220 (46.5%)	191 (63.7%)	
Macular degeneration				
Yes	235 (30.4%)	136 (28.8%)	99 (33.0%)	0.21^b^
No	538 (69.6%)	337 (71.2%)	201 (67.0%)	

^a^ *p* value was calculated with *t*-test.

^b^ *p* values were calculated using the chi-square.

**Table 2:** Comparison of the mean number of treatment injections between PI doses stratified by medication.

Drug (*n* = number of injections)	PI dose (mean ± SD)		*p* value^a,b^
	(*N* = number of subjects)		
	2.5% (*N* = 473)	5% (*N* = 300)	
Aflibercept (A) (*n* = 5099)	5.660 ± 9.146	8.073 ± 10.658	0.004 (0.136)
Bevacizumab (B) (*n* = 1561)	1.789 ± 2.485	2.383 ± 3.771	0.001 (0.321)
Ranibizumab (R) (*n* = 146)	0.121 ± 1.516	0.297 ± 1.915	0.248 (0.342)
Ozurdex (O) (*n* = 554)	0.520 ± 1.871	1.027 ± 3.402	0.023 (0.389)

^a^ *p* values were calculated with negative binomial regression models.

^b^ *p* values in brackets are adjusted for patient's observation time.

**Table 3:** Effect of demographic variables, PI dose, and diagnosis variables on the number of aflibercept injections.^a^

	Negative binomial (unadjusted)			Negative binomial (adjusted)		
	Injection rate ratio	*p* value	95% CI	Injection rate ratio	*p* value	95% CI
Age	1.021	< 0.001	(1.014, 1.028)	1.022	< 0.001	(1.013, 1.030)
Male gender^b^	1.127	0.229	(0.928, 1.368)	1.196	0.068	(0.987, 1.449)
PI_dose^c^	0.861	0.137	(0.707, 1.049)	0.801	0.025	(0.660, 0.972)
Diabetic retinopathy^d^	0.937	0.512	(0.770, 1.139)	2.081	< 0.001	(1.603, 2.701)
Macular degeneration^e^	1.693	< 0.001	(1.380, 2.075)	2.054	< 0.001	(1.543, 2.736)

*Note:* The COVID-19 variable was not included in this model because the time at which the aflibercept injections were administered was unknown.

Abbreviations: CI = confidence interval.

^a^The log of observation time (in days) was included as an offset variable in models.

^b^The binary variable gender was defined as 1 if male or 0 if female.

^c^The binary variable PI dose was defined as 1 for 2.5% or 0 for 5%.

^d^The binary variable was defined as 1 if the patient has a diagnosis of diabetic retinopathy or 0 otherwise.

^e^The binary variable was defined as 1 if the patient has a diagnosis of macular degeneration or 0 otherwise.

**Table 4:** Effect of demographic variables, PI dose, and diagnosis variables on the number of bevacizumab injections.^a^

	Negative binomial (unadjusted)			Negative binomial (adjusted)		
	Injection rate ratio	*p* value	95% CI	Injection rate ratio	*p* value	95% CI
Age	0.974	< 0.001	(0.966, 0.983)	0.974	< 0.001	(0.963, 0.984)
Male gender^b^	1.033	0.819	(0.779, 1.370)	0.950	0.714	(0.719, 1.253)
PI_dose^c^	1.253	0.125	(0.939, 1.672)	1.057	0.706	(0.793, 1.408)
Diabetic retinopathy^d^	1.885	< 0.001	(1.424, 2.495)	1.989	< 0.001	(1.364, 2.900)
Macular degeneration^e^	0.794	0.134	(0.586, 1.074)	1.992	0.001	(1.315, 3.017)

*Note:* The COVID-19 variable was not included in this model because the time at which the bevacizumab injections were administered was unknown.

Abbreviations: CI = confidence interval.

^a^The log of observation time (in days) was included as an offset variable in models.

^b^The binary variable gender was defined as 1 if male or 0 if female.

^c^The binary variable PI dose was defined as 1 for 2.5% or 0 for 5%.

^d^The binary variable was defined as 1 if the patient has a diagnosis of diabetic retinopathy or 0 otherwise.

^e^The binary variable was defined as 1 if the patient has a diagnosis of macular degeneration or 0 otherwise.

**Table 5:** Effect of demographic variables, PI dose, and diagnosis variables on the number of Ozurdex injections.^a^

	Negative binomial (unadjusted)			Negative binomial (adjusted)		
	Injection rate ratio	*p* value	95% CI	Injection rate ratio	*p* value	95% CI
Age	0.987	0.169	(0.969, 1.006)	1.028	0.035	(1.002, 1.054)
Male gender^b^	0.907	0.688	(0.562, 1.463)	0.961	0.902	(0.510, 1.812)
PI_dose^c^	0.809	0.389	(0.499, 1.311)	1.063	0.854	(0.556, 2.032)
Diabetic retinopathy^d^	0.945	0.820	(0.584, 1.532)	0.264	< 0.001	(0.131, 0.533)
Macular degeneration^e^	0.007	< 0.001	(0.002, 0.031)	0.002	< 0.001	(0.0004, 0.011)

*Note:* The COVID-19 variable was not included in this model because the time at which the Ozurdex injections were administered was unknown.

Abbreviations: CI = confidence interval.

^a^The log of observation time (in days) was included as an offset variable in models.

^b^The binary variable gender was defined as 1 if male or 0 if female.

^c^The binary variable PI dose was defined as 1 for 2.5% or 0 for 5%.

^d^The binary variable was defined as 1 if the patient has a diagnosis of diabetic retinopathy or 0 otherwise.

^e^The binary variable was defined as 1 if the patient has a diagnosis of macular degeneration or 0 otherwise.

**Table 6:** Effect of demographic variables, PI dose, diagnosis variables, and COVID-19 pandemic on the total number of injections regardless of product (*N* = 773 patients)^a^.

	Negative binomial (unadjusted)				Negative binomial (adjusted for the other variables)			
	Coefficient	Injection rate ratio	*p* value	95% CI	Coefficient	Injection rate ratio	*p* value	95% CI
COVID-19^b^	−0.159	0.853	0.006	(0.762, 0.955)	0.009	1.009	0.91	(0.867, 1.174)
Male gender^c^	0.073	1.076	0.29	(0.939, 1.233)	0.345	1.412	0.001	(1.152, 1.729)
PI_dose^d^	−0.291	0.748	< 0.001	(0.652, 0.857)	−0.868	0.420	0.005	(0.230, 0.766)
Age	0.014	1.014	< 0.001	(1.009, 1.019)	0.006	1.006	0.11	(0.999, 1.013)
Diabetic retinopathy^e^	−0.173	0.841	0.013	(0.734, 0.964)	0.297	1.346	0.001	(1.135, 1.596)
Macular degeneration^f^	0.459	1.583	< 0.001	(1.376, 1.821)	0.473	1.604	< 0.001	(1.334, 1.929)
PI_dose ∗ age	—	—	—	—	0.008	1.008	0.058	(0.999, 1.017)
COVID-19 ∗ gender	—	—	—	—	−0.320	0.726	0.003	(0.587, 0.898)

Abbreviations: CI = confidence interval.

^a^The log of observation time (in days) was included as an offset variable in models.

^b^The COVID-19 binary variable was defined as 1 if the number of injections was counted after April 9, 2020, or 0 before.

^c^The binary variable gender was defined as 1 if male or 0 if female.

^d^The binary variable PI dose was defined as 1 for 2.5% or 0 for 5%.

^e^The binary variable was defined as 1 if the patient has a diagnosis of diabetic retinopathy or 0 otherwise.

^f^The binary variable was defined as 1 if the patient has a diagnosis of macular degeneration or 0 otherwise.

## Data Availability

The data that support the findings of this study are available from University of Kansas Health System. Restrictions apply to the availability of these data, which were used under license for this study. Data are available from the corresponding author with the permission of University of Kansas Health System.
